# Altered Hippocampal and Striatal Expression of Endothelial Markers and VIP/PACAP Neuropeptides in a Mouse Model of Systemic Lupus Erythematosus

**DOI:** 10.3390/ijms241311118

**Published:** 2023-07-05

**Authors:** Jayden Lee, Sarah Thomas Broome, Margo Iris Jansen, Mawj Mandwie, Grant J. Logan, Rubina Marzagalli, Giuseppe Musumeci, Alessandro Castorina

**Affiliations:** 1 Laboratory of Cellular and Molecular Neuroscience (LCMN), School of Life Science, Faculty of Science, University of Technology Sydney, Sydney, NSW 2007, Australia; jayden.lee2610@gmail.com (J.L.); sarahthomasbroome@gmail.com (S.T.B.); margo.jansen@student.uts.edu.au (M.I.J.); rubina.marzagalli@uts.edu.au (R.M.); 2 Gene Therapy Research Unit, Children’s Medical Research Institute, Faculty of Medicine and Health, The University of Sydney and Sydney Children’s Hospitals Network, Westmead, NSW 2145, Australia; mmandwie@cmri.org.au (M.M.); glogan@cmri.org.au (G.J.L.); 3 Department of Biomedical and Biotechnological Sciences, Anatomy, Histology and Movement Sciences Section, School of Medicine, University of Catania, 95124 Catania, Italy; g.musumeci@unict.it

**Keywords:** neuropsychiatric systemic lupus erythematosus, NZBWF1 mice, autoimmunity, endothelial dysfunction, neuropeptides, PACAP, VIP, striatum, hippocampus

## Abstract

Neuropsychiatric systemic lupus erythematosus (NPSLE) is one of the most common and severe manifestations of lupus; however, its pathogenesis is still poorly understood. While there is sparse evidence suggesting that the ongoing autoimmunity may trigger pathogenic changes to the central nervous system (CNS) microvasculature, culminating in inflammatory/ischemic damage, further evidence is still needed. In this study, we used the spontaneous mouse model of SLE (NZBWF1 mice) to investigate the expression of genes and proteins associated with endothelial (dys)function: tissue and urokinase plasminogen activators (tPA and uPA), intercellular and vascular adhesion molecules 1 (ICAM-1 and VCAM-1), brain derived neurotrophic factor (BDNF), endothelial nitric oxide synthase (eNOS) and Krüppel-like factor 4 (KLF4) and neuroprotection/immune modulation: pituitary adenylate cyclase-activating peptide (PACAP), vasoactive intestinal peptide (VIP), PACAP receptor (PAC1), VIP receptors 1 and 2 (VPAC1 and VPAC2). Analyses were carried out both in the hippocampus and striatum of SLE mice of two different age groups (2 and 7 months old), since age correlates with disease severity. In the hippocampus, we identified a gene/protein expression profile indicative of mild endothelial dysfunction, which increased in severity in aged SLE mice. These alterations were paralleled by moderate alterations in the expression of VIP, PACAP and related receptors. In contrast, we report a robust upregulation of endothelial activation markers in the striatum of both young and aged mice, concurrent with significant induction of the VIP/PACAP system. These data identify molecular signatures of endothelial alterations in the hippocampus and striatum of NZBWF1 mice, which are accompanied by a heightened expression of endogenous protective/immune-modulatory neuropeptides. Collectively, our results support the idea that NPSLE may cause alterations of the CNS micro-vascular compartment that cannot be effectively counteracted by the endogenous activity of the neuropeptides PACAP and VIP.

## 1. Introduction

SLE is an autoimmune disease characterised by the production of autoantibodies that target multiple organ systems [1,2,3]. NPSLE is a common but understudied neurological presentation of SLE [4] that occurs in about 38.3% of SLE patients [5]. Some of the pathogenic features of SLE include vasculitis, vasculopathy and deposition of immune complexes in the brain [1,3]. These pathological events may lead to diffuse cerebral micro-infarctions [6,7] and, consequently, trigger a sequela of clinical manifestations that include repeated bouts of headaches, depressed motor functionality, mood disorders and cognitive dysfunctions [6,8,9]. Over time, NPSLE patients develop life-threatening symptoms, such as seizures and/or stroke [10,11], which contribute to the high mortality rate of afflicted people [12]. Unfortunately, poor prognosis is further aggravated by the limited availability of therapeutic options [13].

It is currently accepted that genetic predisposition, environmental factors and hormonal imbalances collectively contribute to the exacerbation of NPSLE [1,14]. However, the exact causes for the transition from SLE to NPSLE are largely unknown, and disease progression is often unpredictable, with variable neurological manifestations [13]. To improve the prognosis, an early diagnosis and more targeted therapeutical interventions are needed [9]. 

The CNS is able to produce neuropeptides and trophic factors to preserve and protect neurons and glia from damage [15,16]. Pituitary adenylate cyclase-activating polypeptide (PACAP) and vasoactive intestinal peptide (VIP) are two such neuropeptides [17,18]. Their biological activities are mediated by three G protein-coupled receptors, namely, PAC1, VPAC1 and VPAC2 [19,20]. These two peptides, along with their high-affinity receptors, are generally referred to as the PACAP/VIP neuropeptide system [21,22]. This pleiotropic peptidergic system affords neuroprotection and immune modulation in several CNS and non-CNS regions [23,24,25,26], including the vascular compartment [27,28]. Therefore, it is conceivable that the activation of this endogenous protective system is triggered by damage to the brain vasculature, which is severely affected in NPSLE [1,3]. 

Endothelial function is complex, as permeability to solutes must be continually adjusted to the needs of the surrounding microenvironment [29]. A number of enzymes, adhesion molecules, growth factors and transcription factors orchestrate these activities [29,30,31]. Based on the imputed cerebral microvascular involvement in NPSLE pathology, it is possible that alterations in the expression of endothelial markers occur in the CNS of these patients. Interestingly enough—to our knowledge—these pathological domains have been explored only in part in humans or animal models.

Adhesion molecules such as intercellular adhesion molecule-1 (ICAM-1) and vascular cell adhesion molecule-1 (VCAM-1) are responsible for the adhesion of immune cells to the endothelium [32], as well as for the trans-endothelial migration of immune cells to tissues during an inflammatory event [33]. Similarly, Kruppel-like factor 4 (KLF4), a zinc finger-containing transcription factor involved in endothelial cell growth, and endothelial nitric oxide synthase (eNOS) are also upregulated upon vascular inflammation [33,34,35]. 

Plasminogen activators, including the tissue plasminogen activator (tPA) and urokinase-type plasminogen activator (uPA), are proteolytic enzymes with established anti-clotting and angiogenic properties [36,37]. Thrombogenesis is another pathogenic hallmark of NPSLE, likely due to endothelial dysfunction and the consequent increase in platelet activation and aggregation [38]. Therefore, analyses of the expression of endogenous plasminogen activators in the CNS may be useful to assess ongoing vascular damage and intravascular clot formation. Furthermore, tPA cleaves pro-BDNF into the mature and active BDNF [39,40], which can exert its trophic activities and protect the brain from ischemic injury, a direct consequence of CNS thrombosis [41,42].

In the present study, we interrogated two CNS regions that are vulnerable to the effects of NPSLE—the hippocampus and striatum [1,2]—where we investigated the expression of endothelial markers (namely, tPA, uPA, ICAM-1, VCAM-1, BDNF, eNOS and KLF4) and the VIP/PACAP neuropeptide system in a spontaneous animal model of SLE. The goal of this study is to provide molecular insights into the pathogenesis of NPSLE at both early (mild) and late (severe) stages, which will aid in the identification of new therapeutic targets for the treatment of this neurological condition.

## 2. Results

### 2.1. Evidence of Altered Endothelial Markers in the Hippocampus of NZBWF1 Mice

To assess whether NZBWF1 mice exhibited altered expression levels of endothelial markers in brain regions susceptible to the effects of NPSLE (i.e., the hippocampus and striatum) [43,44], we conducted real-time qPCRs and Western blots in mice of two different age groups (2 and 7 mo), as SLE-prone NZBWF1 mice develop clinical features of NPSLE that worsen with age.

**Figure 1:**
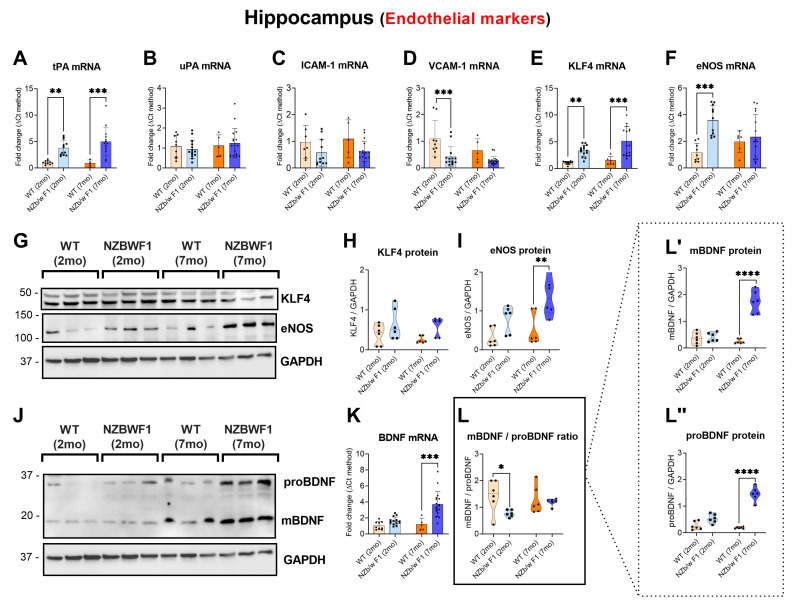
Expression of tPA, uPA, ICAM-1, VCAM-1, KLF4, eNOS and BDNF in the hippocampus of 2- and 7-month-old WT and NZBWF1 mice. Depicted are real-time qPCR analyses of (**A**) tPA, (**B**) uPA, (**C**) ICAM-1, (**D**) VCAM-1, (**E**) KLF4, (**F**) eNOS and (**K**) BDNF gene expression. Relative changes in mRNA levels were determined using the ∆CT method and were normalised to the ribosomal subunit S18 which was used as the housekeeping gene. (**G**,**J**) Representative Western blots and corresponding densitometry of (**H**) KLF4, (**I**) eNOS and (**L**) BDNF protein expression, including both (**L′**) mature BDNF and (**L″**) proBDNF. Densitometric analyses were performed using the ImageJ software ver. 1.53c, and normalised values were determined by dividing the mean optical density of bands over the mean optical density of their corresponding GAPDH bands. Data represent the mean of (*n* = 5) WT 2 mo, (*n* = 7) NZBWF1 2 mo, (*n* = 4) WT 7 mo and (*n* = 9) NZBWF1 7 mo mice in each experimental group. Results are expressed as mean ± S.D. * *p* ≤ 0.05, ** *p* ≤ 0.01, *** *p* ≤ 0.001, **** *p* ≤ 0.0001 vs. WT at the corresponding age, as determined by two-way ANOVA followed by Tukey post hoc test. tPA = tissue plasminogen activator; uPA = urokinase plasminogen activator; ICAM-1 = intercellular adhesion molecule 1; VCAM-1 = vascular cell adhesion molecule 1; KLF4 = Krüppel-like factor 4; eNOS = endothelial nitric oxide synthetase; BDNF = brain-derived neurotrophic factor.

In comparison with aged-matched wild-types (WTs), NZBWF1 mice exhibited a significant increase in hippocampal tPA mRNA expression at both 2 months old (mo) (** *p* ≤ 0.01 vs. WT) and 7 mo (*** *p* ≤ 0.001 vs. WT; Figure 1A). In contrast, neither uPA nor ICAM-1 transcripts differed significantly between the two groups (*p* > 0.05 vs. WT; Figure 1B,C). VCAM-1 mRNA expression was significantly reduced in young (*** *p* ≤ 0.001 vs. 2 mo WTs) but not in old NZBWF1 mice (*p* > 0.05 vs. 7 mo WT; Figure 1D). Similarly to tPA, the expression levels of the transcription factor KLF 4 were robustly increased in both age groups (** *p* ≤ 0.01 vs. 2 mo WT and *** *p* ≤ 0.001 vs. 7 mo WT; Figure 1E), whereas eNOS mRNAs were only significantly higher in the young NZBWF1 group (*** *p* ≤ 0.001 vs. 2 mo WT; Figure 1F).

In contrast to mRNA findings, Western blots showed no significant changes in the expression of KLF4 between WT and NZBWF1 animals, irrespective of age (*p* > 0.05 vs. WT; Figure 1G,H). However, eNOS protein expression was marginally (but not significantly) increased in the hippocampus of young NZBWF1 mice (*p* > 0.05 vs. WTs) and highly significant in older mice (** *p* ≤ 0.01 vs. 7 mo WTs; Figure 1I).

Finally, hippocampal BDNF mRNA expression was not increased in young NZBWF1 mice (*p* > 0.05 vs. 2 mo WT); however, it was remarkably increased in older SLE mice (*** *p* ≤ 0.001 7 mo WTs; Figure 1K). BDNF protein analyses allowed us to discriminate the expression of the cleaved (mature form) BDNF (mBDNF), the uncleaved BDNF precursor (proBDNF) and their relative ratio. In the 2 mo NZBWF1 group, we observed a significant reduction in mBDNF/proBDNF ratio (* *p* ≤ 0.05 vs. 2 mo WT) but no apparent changes in the 7 mo group (*p* > 0.05 vs. 7 mo WT; Figure 1J,L). However, it was observed that the non-significant change in the latter ratio was due to a combined increase of both mBDNF (**** *p* ≤ 0.0001 vs. 7 mo WT; Figure 1L′) and proBDNF (**** *p* ≤ 0.0001 vs. 7 mo WT; Figure 1L″), coherent with the increase seen in transcript levels (Figure 1K). 

### 2.2. Increased Hippocampal Expression of VIP/PACAP and Receptors in Older but Not in Younger NZBWF1 Mice

**Figure 2:**
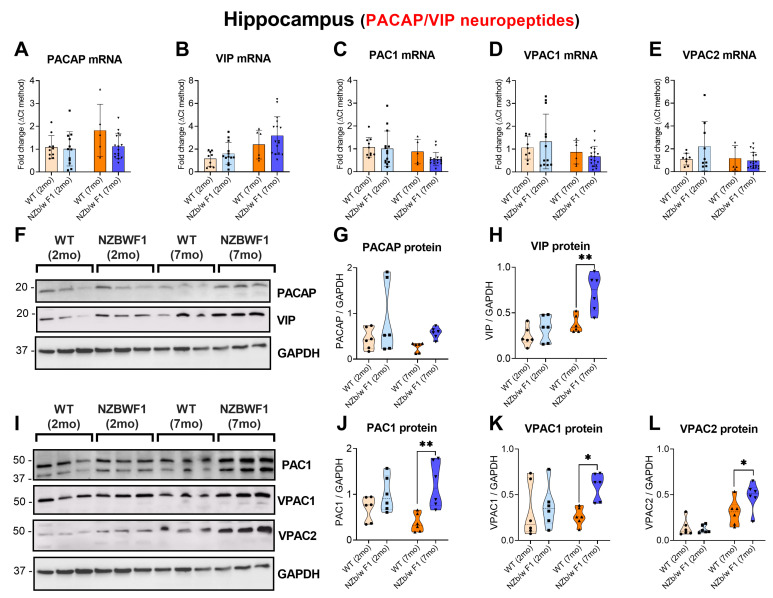
Expression of PACAP, VIP and related receptors, PAC1, VPAC1 and VPAC2 in the hippocampus of 2- and 7-month-old WT and NZBWF1 mice. Depicted are real-time qPCR results of (**A**) PACAP, (**B**) VIP, (**C**) PAC1, (**D**) VPAC1 and (**E**) VPAC2 gene expression. Relative changes in mRNA levels were determined using the ∆CT method and were normalised to the ribosomal subunit S18 which was used as a housekeeping gene. (**F**,**I**) Representative Western blots and related densitometry of (**G**) PACAP, (**H**) VIP, (**J**) PAC1, (**K**) VPAC1 and (**L**) VPAC2 protein expression. Densitometric analyses were performed using the ImageJ software and were normalised to GAPDH bands, here used as loading control. Data represent the mean of (*n* = 5) 2 mo WT, (*n* = 7) 2 mo NZBWF1, (*n* = 4) 7 mo WT and (*n* = 9) 7 mo NZBWF1 mice. Results are expressed as mean ± S.D. * *p* ≤ 0.05 or ** *p* ≤ 0.01 vs. WT at the corresponding age, as determined by two-way ANOVA followed by Tukey post hoc test. PACAP = pituitary adenylate cyclase-activating peptide; VIP = vasoactive intestinal peptide; PAC1 = PACAP receptor 1; VPAC1 = VIP receptor 1; VPAC2 = VIP receptor 2.

Gene expression studies revealed no changes in the expression of the endogenous PACAP or VIP peptides in the hippocampus of NZBWF1 when compared to WTs in either age group (*p* > 0.05 vs. WTs for both PACAP and VIP; Figure 2A,B). Similarly, neither hippocampal PAC1 nor VPAC1/VPAC2 receptors were differentially expressed with respect to aged-matched WTs (*p* > 0.05 vs. WTs for both PAC1 and VPAC1 and VPAC2; Figure 2C–E). In contrast, protein expression studies revealed a mild (though not statistically significant) increase in PACAP protein levels in SLE mice of both age groups (*p* > 0.05 vs. WTs; Figure 2F,G), paralleled by a robust increase in the expression of VIP in 7 mo NZBWF1 mice (** *p* ≤ 0.01 vs. 7 mo WTs; Figure 2H). Likewise, the protein expression of neuropeptides’ receptors was significantly increased in older NZBWF1 mice only (Figure 2I,L). Specifically, we observed a robust PAC1 upregulation (** *p* ≤ 0.01 vs. 7 mo WTs; Figure 2J), along with a significant increase in VPAC1 and VPAC2 expression (* *p* ≤ 0.05 vs. 7 mo WTs; Figure 2K,L).

### 2.3. Robust Transcriptional Alterations of Endothelial Markers in the Striatum of NPSLE Mice

**Figure 3:**
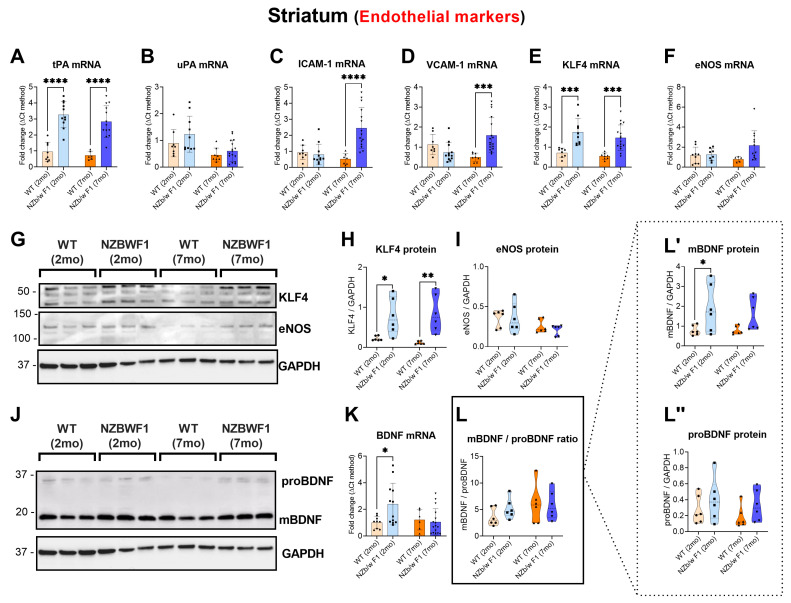
Expression of tPA, uPA, ICAM-1, VCAM-1, KLF4, eNOS and BDNF in the corpus striatum of 2- and 7-month-old WT and NZBWF1 mice. Depicted are real-time qPCR analyses of (**A**) tPA, (**B**) uPA, (**C**) ICAM-1, (**D**) VCAM-1, (**E**) KLF4, (**F**) eNOS and (**K**) BDNF gene expression. Relative changes in mRNA levels were determined using the ∆CT method and were normalised to the ribosomal subunit S18. (**G**,**J**) Representative Western blots and corresponding densitometry of (**H**) KLF4, (**I**) eNOS and (**L**) BDNF protein expression, including both (**L′**) mature BDNF and (**L″**) proBDNF. Densitometric analyses were performed using the ImageJ software, and normalised values were determined by dividing the mean optical density of bands over the mean optical density of their corresponding GAPDH bands. Data represent the mean of (*n* = 5) WT 2 mo, (*n* = 7) NZBWF1 2 mo, (*n* = 4) WT 7 mo and (*n* = 9) NZBWF1 7 mo mice in each experimental group. Results are expressed as mean ± S.D. * *p* ≤ 0.05, ** *p* ≤ 0.01, *** *p* ≤ 0.001, **** *p* ≤ 0.0001 vs. WT at the corresponding age, as determined by one-way ANOVA followed by Tukey post hoc test. tPA = tissue plasminogen activator; uPA = urokinase plasminogen activator; ICAM-1 = intercellular adhesion molecule 1; VCAM-1 = vascular cell adhesion molecule 1; KLF4 = Krüppel-like factor 4; eNOS = endothelial nitric oxide synthetase; BDNF = brain-derived neurotrophic factor.

In the striatum of SLE mice, there was a significant upregulation of tPA mRNA expression in both age groups (**** *p* ≤ 0.0001 vs. WTs; Figure 3A), whereas the expression levels of ICAM-1 and VCAM-1 were only increased in older NZBWF1 mice (ICAM-1: **** *p* ≤ 0.0001 vs. 7 mo WTs; VCAM-1: *** *p* ≤ 0.001 vs. 7 mo WTs; Figure 3C,D). KLF4 gene expression was upregulated in both younger and older mice (*** *p* ≤ 0.001 vs. 2 and 7 mo WTs; Figure 3E). BDNF mRNA expression was significantly increased only in young SLE mice (* *p* ≤ 0.05 vs. 2 mo WTs; Figure 3K). Protein studies confirmed the significant increase in KLF4 expression (* *p* ≤ 0.05 vs. 2 mo WTs and ** *p* ≤ 0.01 vs. 7 mo WTs; Figure 3H). Similarly, the upregulated BDNF transcripts in young NZBWF1 mice were also confirmed at the protein level, with a significant increase in the relative mBDNF protein abundance in these mice (* *p* ≤ 0.05 vs. 2 mo WTs; Figure 3L′).

### 2.4. Striatal Perturbations of VIP/PACAP and Receptors in NZBWF1 Mice

**Figure 4:**
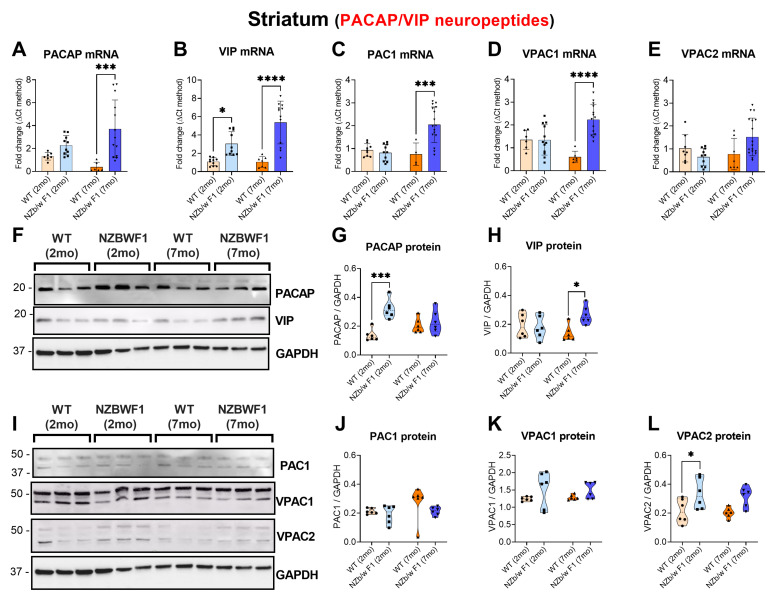
Expression of PACAP, VIP and related receptors, PAC1, VPAC1 and VPAC2 in the corpus striatum of 2- and 7-month-old WT and NZBWF1 mice. Depicted are real-time qPCR results of (**A**) PACAP, (**B**) VIP, (**C**) PAC1, (**D**) VPAC1 and (**E**) VPAC2 gene expression. Relative changes in mRNA levels were determined using the ∆CT method and were normalised to the ribosomal subunit S18 which was used as a housekeeping gene. (**F**,**I**) Representative Western blots and related densitometry of (**G**) PACAP, (**H**) VIP, (**J**) PAC1, (**K**) VPAC1 and (**L**) VPAC2 protein expression. Densitometric analyses were performed using the ImageJ software and were normalised to GAPDH bands, here used as loading control. Data represent the mean of (*n* = 5) 2 mo WT, (*n* = 7) 2 mo NZBWF1, (*n* = 4) 7 mo WT and (*n* = 9) 7 mo NZBWF1 mice. Results are expressed as mean ± S.D. * *p* ≤ 0.05, *** *p* ≤ 0.001 or **** *p* ≤ 0.0001 vs. WT at the corresponding age, as determined by two-way ANOVA followed by Tukey post hoc test. PACAP = pituitary adenylate cyclase-activating peptide; VIP = vasoactive intestinal peptide; PAC1 = PACAP receptor 1; VPAC1 = VIP receptor 1; VPAC2 = VIP receptor 2.

In contrast to what was observed in the hippocampus, transcriptional data pertaining to the neuropeptides and related receptors demonstrated a robust upregulation of gene expression in the striatum of 7 mo NZBWF1 mice (Figure 4A–D), with the exception of VIP, which was also upregulated in younger mice (* *p* < 0.05 vs. 2 mo WT; Figure 4B). Specifically, striatal PACAP (*** *p* ≤ 0.001 vs. 7 mo WTs; Figure 4A), VIP (**** *p* ≤ 0.0001 vs. 7 mo WTs; Figure 4B), PAC1 (*** *p* ≤ 0.001 vs. 7 mo WTs; Figure 4C) and VPAC1 (**** *p* ≤ 0.0001 vs. 7 mo WTs; Figure 4D) mRNAs were all significantly increased. Although not statistically significant, VPAC2 demonstrated an upward trend in mRNA expression (*p* > 0.05 vs. 7 mo WT; Figure 4E). Protein expression data only in part confirmed our transcriptional observations. PACAP expression was increased in younger SLE mice only (*** *p* ≤ 0.001 vs. 2 mo WTs; Figure 4G), whereas VIP increased only in older SLE mice (* *p* ≤ 0.05 7 mo WTs; Figure 4H). Again, VPAC2 expression increased in younger SLE mice (* *p* ≤ 0.05 vs. 2 mo WTs; Figure 4L), while PAC1 and VPAC1 were unaffected (*p* > 0.05 vs. WTs; Figure 4J,K).

## 3. Discussion

In this study, we demonstrate for the first time that in spontaneous SLE mice (NZBWF1), brain regions highly susceptible to the longstanding detrimental effects of SLE—such as the hippocampus and striatum—show molecular signatures of cerebrovascular pathology. We also provide evidence that the age-related increase in disease severity in SLE mice shows a relationship with the increased expression of endogenous protective neuropeptides. 

In line with the idea of CNS perturbations at various neurovascular interfaces in NPSLE patients [45], our data identified a number of alterations of genes/proteins associated with cerebrovascular health in our SLE model, which cannot be simplified by a generic inflammatory response within the CNS [32,33,34,46,47]. In fact, the combined upregulation of proteolytic enzymes (tPA), adhesion molecules (ICAM-1 and VCAM-1), vasodilator mediators (eNOS), regulators of vascular integrity (KLF-4) [48] and trophic factors (proBDNF and mature BDNF) [49] corroborates the idea of a specific cerebrovascular involvement. 

Analyses of endothelial markers revealed stronger molecular signs of vascular alterations in the striatum when compared with the hippocampus. This is in apparent contradiction with the literature, as the hippocampus is known to be more vulnerable to inflammatory insults [50,51]. Nonetheless, at least two independent clinical MRI imaging reports highlighted the corpus striatum as a CNS structure susceptible to the deleterious effects of SLE in an NPSLE form referred to as striatal dominant lupus encephalitis [52,53]. Although the underlying reasons for the higher vulnerability of the striatum in this subset of NPSLE patients remain obscure, these preclinical observations pinpoint the prominent involvement of this brain structure in the pathophysiology of the disease. 

Neurons represent the main source of BDNF in the CNS, especially when it is under the threat of pathogens or in response to trauma [47,54]. However, cerebral endothelial cells also produce the bioactive form of this critical neurotrophic factor [55]. Here, we found that BDNF mRNAs were upregulated both in the hippocampus and striatum of SLE mice. Still, we observed distinct spatiotemporal differences in the regulation of BDNF gene and protein expression in these two CNS structures. Specifically, in the hippocampus, transcripts were significantly upregulated only in NZBWF1 mice with severe disease (7 mo) but not in the milder form (2 mo), and these data were consistent with protein expression changes of both proBDNF and its cleaved bioactive product, mBDNF. Conversely, striatal BDNF transcripts were upregulated only in mice with a milder form of SLE (2 mo) and not in the severe form (7 mo) of the disease. These results were corroborated by similar protein expression changes of mBDNF but not proBDNF (unchanged at both ages). We interpreted these spatial (i.e., striatum vs. hippocampus) and temporal differences (i.e., 2 vs. 7 mo) in BDNF profiles as indirect correlates of the different degrees and timing of vascular pathology progression in our SLE model. However, further and more direct evidence will be needed to provide a causative link that will confirm such observations.

Our study also identified a possible link between eNOS and BDNF expression levels in the hippocampus but not in the striatum. This association is of particular interest, as it suggests the coexistence of both vascular and ischemic events in this vulnerable CNS region. The hippocampus is notoriously vulnerable to ischemic damage [56,57], and there have been suggestions of a possible connection between cerebrovascular damage, ischemia and the cognitive decline seen in people with NPSLE [58]. The endothelial enzyme (eNOS) is responsible for the production of nitric oxide (NO) by endothelial cells [59], whose increase in the CNS promotes BDNF induction in an attempt to protect neurons from ischemic damage [60,61]. Therefore, based on our findings and in view of the previously reported heightened thrombogenic activity in NPSLE patients [38,62], it is possible that the combined upregulation of hippocampal eNOS and BDNF could be interpreted as evidence of ischemia secondary to micro- and macro-vascular alterations in the hippocampus of NZBWF1 animals. 

Upon comparing the expression of each member of the PACAP/VIP neuropeptide system in this preclinical model of SLE, we observed a global upregulation of the PACAP, VIP and receptors both in the hippocampus and striatum, which was more remarkable (at the protein level) in the hippocampus of mice with severe disease (older mice). In view of the well-known involvement of these neuropeptides as neuroprotective and immune-modulatory agents [24,26], these results imply the activation of endogenous homeostatic mechanisms in an attempt to reduce the damage occurring in vulnerable brain regions. Intriguingly, our analyses also revealed that VPAC2 receptor gene expression was the least to be affected. Literature reports that VPAC2 expression is normally upregulated in response to CNS inflammation, whereas PAC1 and VPAC1 are more responsive to stress and/or other types of neuronal injury [63]. Therefore, although CNS inflammation is thought to contribute to NPSLE pathogenesis [64], our evidence suggests that inflammation, at least in the CNS areas investigated, may not be prominent enough to induce significant VPAC2 induction. Alternatively, it is possible that VPAC2 mRNA expression may simply not be upregulated at the specific ages we tested the mice, in view of the dynamic nature at which gene expression changes occur. Furthermore, VPAC2 protein expression did not corroborate our transcriptional findings, hence showing early upregulation in the striatum of 2-month-old mice (but not in older mice) and the opposite in the hippocampus, where upregulation was seen in 7-month-old mice only. Combined, these observations define specific spatiotemporal changes in VPAC2 expression in SLE mice.

We also observed some unique differences in the transcriptional and post-transcriptional regulation of peptides and receptors in the two investigated CNS regions. For instance, mRNA data showed no transcriptional differences between WTs and NZBWF1 animals in the hippocampus, irrespective of age (hence disease severity). However, protein expression studies revealed robust increases in the expression of VIP, PAC1 and VPAC1 receptors in older mice. While it is accepted that mRNA and protein findings often differ, the remarkable difference suggests that the regulatory activities on the hippocampal PACAP/VIP system of SLE mice are likely to be mainly post-transcriptional. This observation agrees with previous studies for our laboratory in a different mouse model, where hippocampal changes of PACAP/VIP were observed mainly at the protein level, rather than at transcriptional levels [65]. Surprisingly, these findings are in sharp contrast with the results seen in the striatum. In fact, most of the striatal changes in the expression of PACAP/VIP family members occurred at the transcriptional level, with fewer changes at the protein level. The reason for such a difference is not clear. Although there is some evidence portraying CNS regional-specific differences in gene expression and promoter usage in the aging human brain [66], to our knowledge, there is no prior data to explain such remarkable (post)transcriptional differences between the two structures in the mouse brain, at least with regard to PACAP/VIP neuropeptides regulatory activities. Specific differences in the vascular basement membranes and their functionalities between the hippocampus and striatum as well as differing parenchymal composition should be accounted for these differences, as these structural dissimilarities have been previously reported in the cerebral vasculature of aging mice [67]. However, it should be noted that striatal expression of VIP, PAC1 and VPAC2 proteins was consistently lower than in the hippocampus, so we cannot exclude that the reduced protein abundance in this brain structure may have interfered with the resolution of our semi-quantitative determinations.

In conclusion, the data provided in this study highlight specific molecular alterations that are highly suggestive of vascular involvement in the selected brain regions of SLE mice. These pathological processes seem to induce the activation of the PACAP/VIP neuroprotective system, perhaps as a homeostatic mechanism to hinder neuronal damage in affected regions. Future work is warranted to determine the existence of clinical correlates with the observed molecular changes. However, despite further clinical and morphological work is needed to confirm these findings, collectively our data add novel molecular evidence that strengthens the emerging theory of autoimmune-mediated cerebrovascular impairment in NPSLE. Our results also suggest that protective neuropeptides and trophic factors, such as PACAP, VIP or BDNF, may be considered as viable therapeutic targets to ameliorate the CNS damage caused by this debilitating form of SLE.

## 4. Materials and Methods

### 4.1. Animals

All animal care and experimental procedures were evaluated and approved by the Children’s Medical Research Institute (CMRI)/Children’s Hospital at Westmead (CHW) Animal Care and Ethics Committee (Protocol C351, approved on 5 August 2016). All experiments were conducted in agreement with the Australian Code of Practice for the Care and Use of Animals for Scientific Purposes. All NZBWF1 and BALB/c mice used for this study were obtained from the Jackson Laboratory (Bar Harbor, ME). For convenience, age-matched BALB/c mice (used in this study as controls) will be referred to as wild-type (WT) [68]. Mice received standard rodent chow and water ad libitum and were housed in standard cages for the duration of the experiments. Both WT and aged-matched NZBWF1 mice were sacrificed via CO_2_ asphyxiation followed by cardiac puncture. The cohort of mice used for this study included WT 2 months old ((mo), *n* = 5), NZBWF1 2 mo (*n* = 7), WT 7 mo (*n* = 4) and NZBWF1 7 mo (*n* = 9).

### 4.2. Brain Micro-Dissections

Following euthanasia, whole brains from NZBWF1 mice and age-matched BALB/c controls (2 and 7 mo, respectively) were collected, washed in ice-cold PBS and immediately submerged in RNAlater stabilisation solution (Thermo Fisher, Sydney, NSW, Australia, #AM7021) prior to snap freezing in liquid nitrogen. At the time of dissections, brains were placed on a sterile, ice-cold PCR-clean surface and micro-dissected under a stereoscopic microscope (10× magnification), using the mouse brain atlas as a reference [69]. Brain tissue blocks encompassing the entire hippocampus (both dorsal and ventral portion) and the entire corpus striatum were excised and stored at −80 °C until downstream RNA and protein extraction.

### 4.3. RNA Extraction, cDNA Synthesis and Real-Time Quantitative Polymerase Chain Reaction (RT-qPCR)

RNA was extracted from the hippocampus and striatum of both 2 and 7 mo WT and SLE mice via homogenisation in Tri-Reagent (Sigma-Aldrich, Castle Hill, NSW, Australia). Samples were incubated at room temperature for 5 min in Tri-Reagent before 200 µL of chloroform was added. Samples were then vortexed and incubated for 3 min at room temperature and subsequently centrifuged at 12,000× *g* for 15 min at 4 °C. The upper aqueous phase containing RNA was collected and placed in new tubes. RNA was then precipitated using 500 µL of isopropyl alcohol (2-propanol) (Sigma-Aldrich, Castle Hill, NSW, Australia). Samples were mixed and incubated for 10 min at room temperature, followed by a second centrifugation step at 12,000× *g* for 10 min at 4 °C. The precipitated RNA pellet was washed three times with 75% ethanol and air-dried. RNA concentrations were determined using NanoDrop^TM^ 2000 (Thermo Fisher Scientific, Scoresby, VIC, Australia). About 1 µg of total RNA was used to synthesise cDNA using the Tetro cDNA Synthesis Kit (Bioline, Narellan, NSW, Australia). Real-time qPCR was performed by adding the following to each well of the 96-well PCR plate: 0.4 µL of Milli-Q water, 3 µL cDNA, 5 µL iTaq Universal SYBR Green PCR Master Mix (Bio-Rad, South Granville, NSW, Australia) and 0.8 µL of the forward and reverse primers (5 µM) for the gene of interest (primers used in this study are listed in Table 1). Detection was performed using the CFX96 Touch TM Real-Time PCR Detection System (Bio-Rad, NSW, Australia). Instrument settings were as follows: (1) 95 °C for 2 min, (2) 60 °C for 10 s, (3) 72 °C for 10 s, (4) plate read and (5) repeat steps 2–4 for 45 cycles. With regard to melting curve analyses, settings were as follows: (1) 65 °C for 35 s, (2) plate read and (3) repeat steps 1–2 for 60 cycles. To determine expression changes of mRNA, the mean fold change values of each sample were calculated using the ΔCt method, as previously described by Schmittgen and Livak [70] using S18 as the reference gene [71]. PCR product specificity was confirmed by melting curve analysis, with each gene demonstrating a single peak.

### 4.4. Protein Extraction and Western Blot

Protein was extracted from the brains of both 2 and 7 mo WT and NZBWF1 mice via homogenisation in ice-cold radioimmunoprecipitation assay (RIPA) buffer, supplemented with a protease inhibitor cocktail (cOmplete, Mini, EDTA-free Protease Inhibitor Cocktail, Sigma-Aldrich, Castle Hill, NSW, Australia). RIPA was added at a 1:10 *w*/*v* ratio. Samples were sonicated for 10 s, 3× times, with 30 s intervals (on ice) between each sonication. Homogenates were then centrifuged for 10 min at 12,000× *g* and 4 °C, and the supernatant (containing the protein) was retained. Protein quantification was performed using bicinchoninic acid assay (Pierce BCA Protein Assay Kit, Thermo Fisher Scientific, VIC, Australia). Absorbance measurements were recorded at 562 nm on a microplate reader. Thirty micrograms of protein lysates from each sample were resolved using sodium dodecyl sulphate-polyacrylamide gel electrophoresis (SDS-PAGE) using 4–20% Mini-PROTEAN TGX Stain-Free Gels (15-wells, Bio-Rad, VIC, Australia). Proteins were then transferred to a PVDF membrane using the semi-dry method (Bio-Rad, Trans-Blot Turbo Transfer System). Incubation in primary antibody was performed overnight in 5% skim milk and TBST blocking solution at 4 °C on a slow oscillation of 50–60 rpm. Secondary antibody incubation was then performed for 1 h at room temperature (primary antibodies and related dilutions are listed in Table 2). Blots were visualised using the chemiluminescence Bio-Rad Clarity Western ECL Blotting Substrate Solution (Bio-Rad, VIC, Australia), and images were obtained using the Bio-Rad ChemiDoc MP System (Bio-Rad, VIC, Australia). Densitometric analyses of protein bands were conducted using NIH ImageJ software, and ratios were normalised to GAPDH, which was used as a loading control [72].

### 4.5. Statistical Analyses

All experimental data were reported as mean ± S.D. Gene and protein expression data were analysed using GraphPad Prism software version 9.02 for Windows (GraphPad Software, San Diego, CA, USA). Normality tests were performed using the Kolmogorov–Smirnov test. Upon confirmation of normal distribution of data, comparisons between groups were analysed factoring age (2 vs. 7 mo) and genotypes (WT vs. NZBWF1) using two-way ANOVA, followed by Tukey post hoc tests, unless otherwise indicated. *p*-values ≤ 0.05 were considered statistically significant. 

## Figures and Tables

**Table 1:** List of primers used in real-time qPCR analyses.

Accession No.	Gene Name (in Parenthesis)	Primer Sequences (5′-3′)	Length (bp)
NM_009625.2	Pituitary adenylate cyclase-activating polypeptide (*Adcyap1*)	Fwd CTGCGTGACGCTTACGCCCT Rev CCTAGGTTCTCCCCCGCGCC	152
NM_011702.2	Vasoactive intestinal peptide (*Vip*)	Fwd TGGCAAACGAATCAGCAGCAGCA Rev AGCCATTTGCTTTCTGAGGCGGG	106
NM_007407.3	PAC1 receptor (*Adcyap1r1*)	Fwd CAGTCCCCAGACATGGGAGGCA Rev AGCGGGCCAGCCGTAGAGTA	139
NM_011703.4	VPAC1 receptor (*Vipr1*)	Fwd TCAATGGCGAGGTGCAGGCAG Rev TGTGTGCTGCACGAGACGCC	127
NM_009511.2	VPAC2 receptor (*Vipr2*)	Fwd GCGTCGGTGGTGCTGACCTG Rev ACACCGCTGCAGGCTCTCTGAT	155
NM_008872.2	Tissue plasminogen activator (*Plat*)	Fwd GCCTGTCCGAAGTTGCAGCGA Rev TGCTGTGCTCCACGTGCCTC	184
NM_008873.3	Urokinase plasminogen activator (*Plau*)	Fwd TTCGCAGCCATCTACCAGAA Rev TGGGAGTTGAATGAAGCAGTG	117
NM_010493.3	Intercellular adhesion molecule-1 (*Icam 1*)	Fwd CCTCCGGACTTTCGATCTTC Rev TCACTGCTGTTTGTGCTCTC	180
NM_011693.3	Vascular cell adhesion molecule-1 (*Vcam 1*)	Fwd GGATACTGTTTGCAGTCTCTCA Rev GCGTTTAGTGGGCTGTCTAT	160
NM_007540.4	Brain-derived neurotrophic factor (*Bdnf*)	Fwd CGAGTGGGTCACAGCGGCAG Rev GCCCCTGCAGCCTTCCTTGG	160
NM_008713.4	Endothelial nitric oxide synthase (*Nos3*)	Fwd AGGTATTTGATGCTCGGGAC Rev CTGTGATGGCTGAACGAAGA	108
NM_010637.3	Krüppel-like factor 4 (*Klf4*)	Fwd CCCACACTTGTGACTATGCAG Rev GTTTCTCGCCTGTGTGAGTT	90
NM_011296.2	18s ribosomal subunit (*s18*)	Fwd CCCTGAGAAGTTCCAGCACA Rev GGTGAGGTCGATGTCTGCTT	145

Forward and reverse primers were selected from the 5′ and 3′ regions of each gene’s mRNA sequence. The expected length of each amplicon is indicated in the right column.

**Table 2:** List of primary antibodies used for Western blot analyses.

Primary Antibodies (All Raised in Rabbit)	Dilution	Source (Cat. No.)
Pituitary adenylate cyclase-activating polypeptide (PACAP)	1:1000	GeneTex (Irvine, CA, USA, GTX37576)
Vasoactive intestinal peptide (VIP)	1:1000	GeneTex (Irvine, CA, USA, GTX129461)
PAC1 receptor	1:1000	GeneTex (Irvine, CA, USA, GTX30026)
VPAC1 receptor	1:500	Sigma-Aldrich (Castle Hill, NSW, Australia, SAB4503084)
VPAC2 receptor	1:500	Sigma-Aldrich (Castle Hill, NSW, Australia, AB2266)
Brain-derived neurotrophic factor (BDNF)	1:1000	GeneTex (Irvine, CA, USA, GTX132621)
Endothelial nitric oxide synthase (eNOS)	1:1000	GeneTex (Irvine, CA, USA, GTX129058)
Krüppel-like factor 4 (KLF4)	1:1000	GeneTex (Irvine, CA, USA, GTX101508)
Glyceraldehyde-3-phosphate dehydrogenase (GAPDH)	1:2000	Bio-Rad (Gladesville, NSW, Australia, VPA00187)

## Data Availability

All data are reported in the published version of this article. Raw data can be made available upon reasonable request to authors.
